# Supplementary material for: Avoiding misleading estimates using mtDNA heteroplasmy statistics to study bottleneck size and selection
Source: G3 (Bethesda). 2023 Mar 23;13(6):jkad068. doi: 10.1093/g3journal/jkad068 (PMC10234379; doi:10.1093/g3journal/jkad068)
Supplement: jkad068_Supplementary_Data [file jkad068_supplementary_data.pdf]

Avoiding misleading estimates using mtDNA heteroplasmy statistics to study bottleneck size and selection

Supplementary Material

Theoretical examples

Consider taking two heteroplasmy measurements in two samples, and observing one homoplasmic wildtype and one homoplasmic mutant $\underline{h} = (0, 1)$. The sample variance is:

$$s^2 = 1/(n-1)\ \Sigma\ (h - \hbar)^2$$
$$= 1 \times (0.25 + 0.25) = 0.5$$
$$\hat{n}_b = \hbar(1-\hbar)\ /\ s^2$$
$$= 0.5$$

Leaving us with a bottleneck size of 0.5 segregating units. Following this example, to estimate parameters of the Kimura distribution with the method of moments, we would use:

$$\mu = p$$
$$\sigma^2 = p\,(1-p)(1-b)$$
$$b = 1 - \sigma^2\ /\ (p\ (1-p))$$

so for our (0, 1) sample, if we use the sample mean $\hbar = 0.5$ as an estimate for population mean p and the sample variance $s^2 = 0.5$ as an estimate for population variance σ^2, we estimate the nonsensical $b = 1 – 0.5 / 0.25 = -1$.

An alternative, maximum likelihood fit gives us $p = 0.5$ (95% c.i.s 0.058-0.941), and a point estimate of $b \simeq 0$ (and corresponding $n = 1$) – all readily interpretable as well-behaved parameters of the Kimura distribution. Confidence intervals on n_b can be estimated via taking likelihood derivatives or resampling, the latter of which is better behaved for this small, extreme dataset (see below), and gives n_b between 1 and 13.6 with 95% confidence.

Numerical issues

An issue can arise when numerically maximising the likelihood associated with homoplasmic data. Because the parameter b is constrained to lie on the interval [0,1], we either bound the domain of optimisation or use a transformation to cast the real line onto that interval. In both cases, the gradients in the associated Hessian matrix behave poorly numerically when b approaches 0 – as is the case for homoplasmic data, where $b = 0$ is the point estimate. In these cases, we found that using bootstrap resampling to characterise confidence intervals for b was the better approach. Of course, bootstrapping could be used for confidence intervals in non-homoplasmic cases too, but for reasons of computational speed and accuracy we suggest Fisher information for estimating p in all cases and for estimating b in cases of heteroplasmy, and bootstrapping for estimating b in cases of homoplasmy.

Another numerical issue involves minimising the KS distance over parameterisations. Because the KS distance surface as a function of parameter p and b can be non-convex (Fig. 1Aiii), it is hard to guarantee that the best solution has been found: the local optimum found may depend on initial conditions. In our implementation we perform two searches,

using the method-of-moments and maximum likelihood parameter estimates as two different initial conditions, and reporting the best solution found from these two searches. We found this to generally identify good solutions based on exhaustively visualising the full surface.

Kimura fourth central moment

The original article from Kimura (1955) gives an expression for the *n*th moment about zero of the Kimura distribution:

$\mu'_n = p + \Sigma_i (2i+1)\, p(1-p)\, (-1)^i\, {}_2F_1(1-i, i+2, 2, p)\, b^{i(i+1)/2}\, n!\, (n-1)! / (\, (n+i)!\, (n-i-1)!\,)$

where we have used the $b = \exp(-t / N_{eff})$ substitution from Wonnapinij *et al*. (2008).

The infinite sums in this expression terminate when the numerator in the summand contains a factor of zero, hence when $i \geq n$. The highest i value we need to consider is i = 4, and for these values the hypergeometric function takes simple polynomial forms: ${}_2F_1(1-i, i+2, 2, p) = \{1, 1-2p, 1-5p+5p^2, 1-9p+21p^2-14p^3\}$ respectively for $i = \{1, 2, 3, 4\}$.

Using these expressions, the non-central moments are then
$\mu'_2 = p + b\, p(p-1)$
$\mu'_3 = p + b\, p(p-1)\, (3 + b^2(2p-1)) / 2$
$\mu'_4 = p + b\, p(p-1)\, (9 + 5b^2(2p-1) + b^5(5p^2 - 5p + 1))/5$

Central moments are related to moments about zero by a family of expressions, of which the fourth is
$\mu_4 = \mu'_4 - 4\, \mu\, \mu'_3 + 6\, \mu^2\, \mu'_2 - 3\, \mu^4$

For the fourth central moment we therefore have
$\mu_4 = p + b\, p(p-1)\, (9 + 5b^2(2p-1) + b^5(5p^2 - 5p + 1))/5 - 4\, p\, (p + b\, p(p-1)\, (3 + b^2(2p-1)) / 2) + 6\, p^2\, (p + b\, p(p-1)) - 3\, p^4$

Which is equivalent to the Wonnapinij *et al*. (2010) expression with the identity $\sigma^2 = p(1-p)(1-b)$.

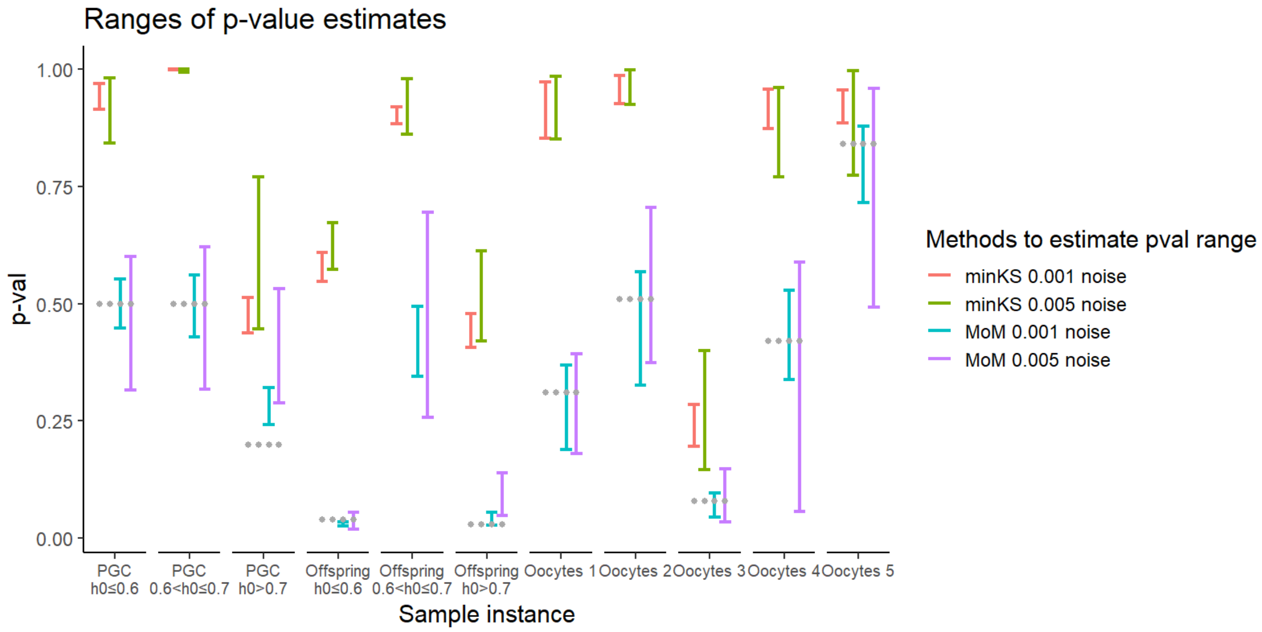

Supplementary Fig. A. **Influence of small observation perturbations on reported p-values from the KS test.** Ranges of estimated p-values from the WCS-K approach, with small amounts of noise added to given observations. Originally reported p-values are given by grey dots. MoM, method-of-moments fitting (as is typically used); minKS, minimum KS distance fitting. Even small amounts of noise in reported observations (much less than 1%) dramatically expand the range of p-values that can arise from the WCS-K approach. MinKS fits, providing better fits to the Kimura distribution than MoM fits, universally give higher p-values.

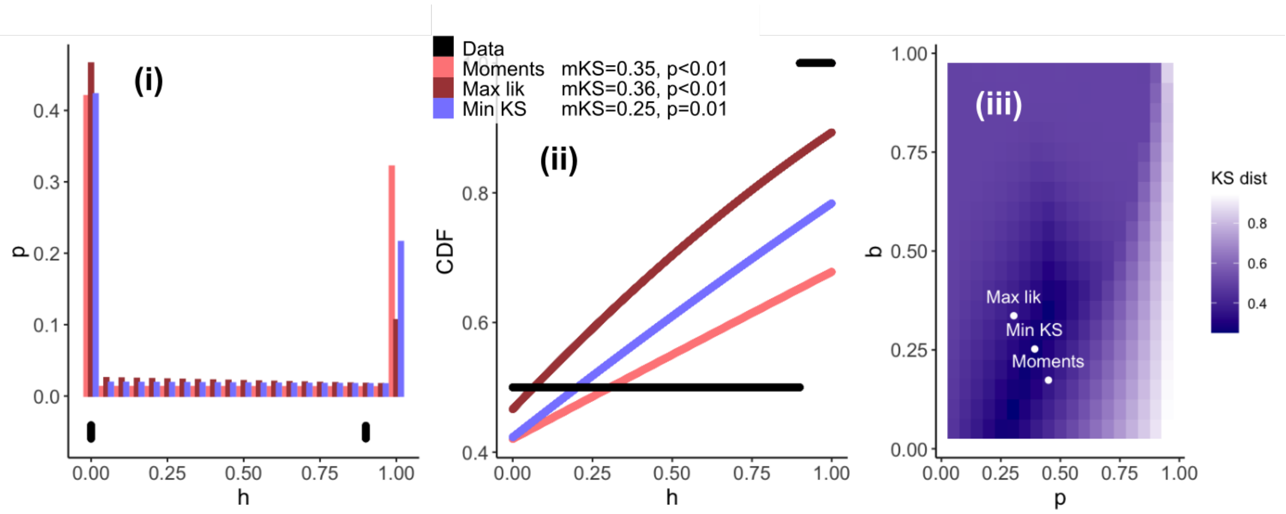

Supplementary Figure B. **Example of synthetic data that significantly departs from the Kimura distribution.** The heteroplasmy measurements consist of the pair (0,0.9) repeated 50 times. As in Fig 1, (i) probability distributions for each fit; (ii) comparison of cumulative distribution functions (CDFs) from the data and for each fit; (iii) the KS distance between empirical observations and theoretical distribution with parameters of the Kimura distribution. mKS, KS distance for each method of fitting; p-values are from the Monte Carlo KS test in WCS-K. As the sample size is higher in this case, the fit using the method of moments and that using maximum likelihood provide more similar parameter estimates.

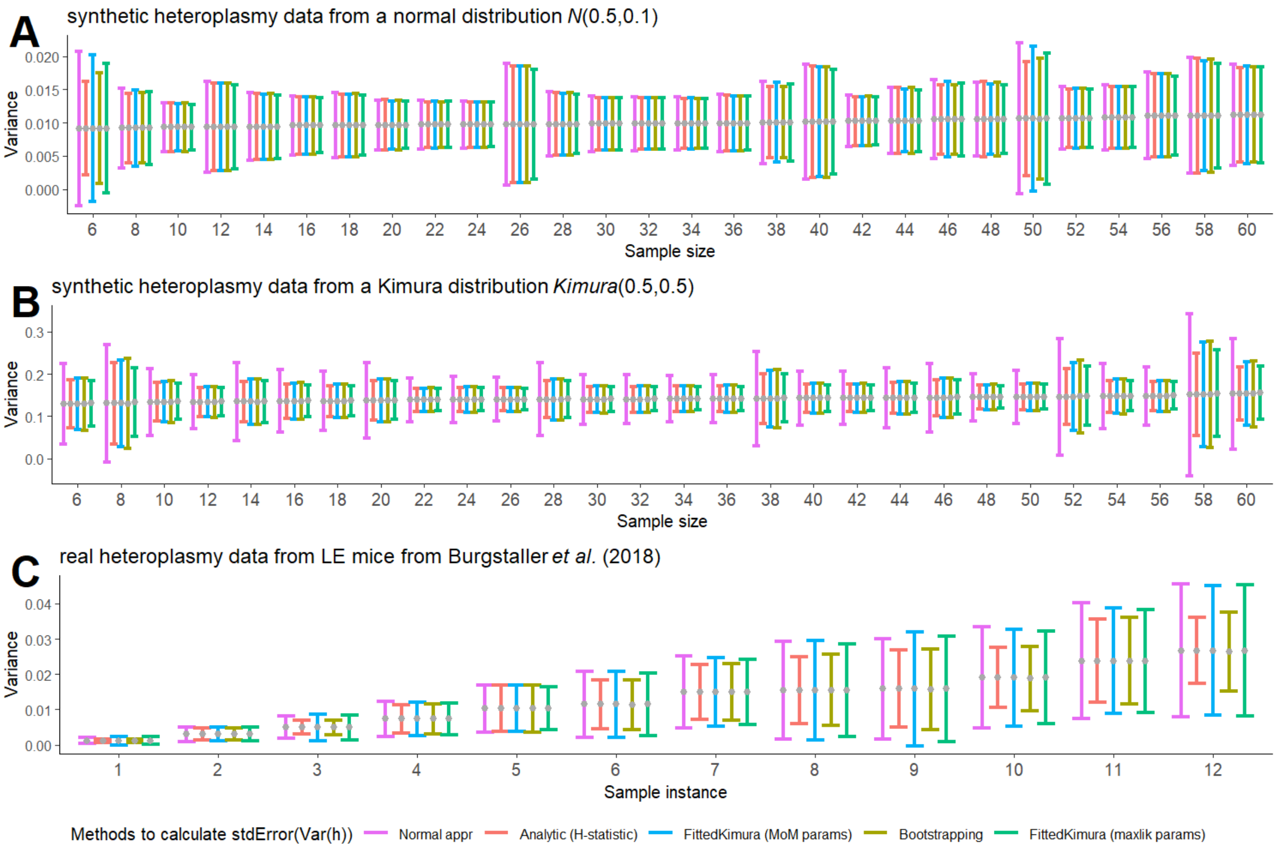

Supplementary Figure C. **Different ways to estimate the uncertainty of variance in samples from (A) synthetic normal distribution; (B) synthetic Kimura distribution; (C) example mouse data.** For the synthetic distributions, samples are ordered by sample size; for the mouse data, 12 example LE type samples are depicted for which the ordering is (for just visual clarity) ascending according to the mean sample variance. Error bars correspond to 2x the standard error of variance as calculated by each method. Analytic (h-statistic) and bootstrapped results are typically comparable; Kimura fits often provide a more conservative estimate of V(h) variance.

References

Burgstaller, J.P., Kolbe, T., Havlicek, V., Hembach, S., Poulton, J., Piálek, J., Steinborn, R., Rülicke, T., Brem, G., Jones, N.S. and Johnston, I.G., 2018. Large-scale genetic analysis reveals mammalian mtDNA heteroplasmy dynamics and variance increase through lifetimes and generations. *Nature communications*, *9*(1), p.2488.

Kimura, M., 1955. Solution of a process of random genetic drift with a continuous model. *Proceedings of the National Academy of Sciences*, *41*(3), pp.144-150.

Wonnapinij, P., Chinnery, P.F. and Samuels, D.C., 2008. The distribution of mitochondrial DNA heteroplasmy due to random genetic drift. *The American Journal of Human Genetics*, *83*(5), pp.582-593.

Wonnapinij, P., Chinnery, P.F. and Samuels, D.C., 2010. Previous estimates of mitochondrial DNA mutation level variance did not account for sampling error: comparing the mtDNA genetic bottleneck in mice and humans. *The American Journal of Human Genetics*, *86*(4), pp.540-550.
